# Prevalence of Malnutrition in a Group of Institutionalized Psychogeriatric Patients Using Different Diagnostic Criteria

**DOI:** 10.3390/nu16081116

**Published:** 2024-04-10

**Authors:** Beatriz de Mateo Silleras, Sara Barrera Ortega, Laura Carreño Enciso, Sandra de la Cruz Marcos, Paz Redondo del Río

**Affiliations:** 1 Department of Nutrition and Food Science, Faculty of Medicine, University of Valladolid, 47005 Valladolid, Spain; bmateo@uva.es (B.d.M.S.); laura.carreno@uva.es (L.C.E.); paz.redondo@uva.es (P.R.d.R.); 2 Spanish Society of Community Nutrition (SENC), 08029 Barcelona, Spain; 3 Psycho-Geriatric Area, Assistance Center of San Juan de Dios, 34005 Palencia, Spain; sara.barrera@sjd.es

**Keywords:** malnutrition, GLIM criteria, ESPEN criteria, bioelectrical impedance, phase angle, elderly, psychogeriatric patients

## Abstract

Malnutrition (MN) is a highly prevalent condition in the elderly. It is associated with functional impairment, disability, frailty, and sarcopenia. The aim was to analyze the capacity of GLIM and ESPEN criteria to diagnose MN in a sample of institutionalized psychogeriatric patients. Clinical and anthropometric data were collected in a cross-sectional study. Patients’ frailty, dependence, functional capacity, MNA, hand-grip strength (HS), and sarcopenia were evaluated. Body composition (BC) was estimated by conventional bioimpedance analysis. MN diagnosis was established using the ESPEN and the GLIM criteria based on fat-free mass index (GLIM-FFMI), appendicular skeletal muscle mass index (GLIM-ASMMI), skeletal muscle mass index (GLIM-SMMI), and HS (mGLIM). Ninety-two patients (57.6% men; mean age: 79.4 years) were studied. Depending on the diagnosis criteria, MN prevalence was between 25% (ESPEN) and 41.3% (GLIM-SMMI). Agreement between ESPEN and all GLIM criteria was poor, but it was excellent between all GLIM criteria (kappa > 0.8). Phenotypic criteria carried more weight in the diagnosis of MN than etiological ones. Depending on the parameter used, the prevalence of reduced muscle mass was notably different. Differences in BMI, BC, inflammation, and albumin are detected by the GLIM-FFMI criteria in the MN and non-MN subjects. Also, this criterion is the only one that identified differences in phase angle (PhA) between these groups. In the elderly, PhA can be very useful to monitor nutritional status.

## 1. Introduction

Malnutrition (MN) is a highly prevalent condition in the elderly. It is associated with functional impairment, disability, frailty, sarcopenia, reduced quality of life, and increased morbidity and mortality [1,2,3,4]. In institutionalized older adults, it has also been associated with lower physical performance, muscle mass and strength, cognitive ability, and increased risk of depression [5].

MN is common in older adults with dementia. It has been related to their cognitive, metabolic, and neurochemical status and to other conditions derived from aging [6,7,8]. The prevalence of MN and risk of MN in institutionalized older adults with dementia has been recently documented by several systematic reviews and meta-analyses. Evidence shows that, depending on the populations and tools used for diagnosis, the prevalence is up to 32.52% and 57.43%, respectively [7]. Another mental condition that has demonstrated a relation with nutritional status is schizophrenia. It has been associated with both weight gain (overweight and obesity) and malnutrition states due to behavioral issues and an inadequate diet and lifestyle [9].

There are several different diagnostic criteria for MN detection. Since, to date, no consensus has been reached on which of them is most appropriate, it is difficult to compare the results obtained in different studies regarding the prevalence of MN and the effectiveness of nutritional therapies.

In 2015, the European Society for Clinical Nutrition and Metabolism (ESPEN) published a “consensus for simple, clear and generally applicable diagnostic criteria of malnutrition”. The aim was “to provide criteria that are independent of etiologic mechanisms and that can be used for all patients and in all clinical settings” [10]. These criteria identify MN whenever BMI is less than 18.5 kg/m^2^ or when any of the following characteristics are met: unintentional weight loss >10% over an indefinite time or >5% over the last 3 months combined with either (i) body mass index (BMI) <20 kg/m^2^ if <70 years of age, or <22 kg/m^2^ if ≥70 years of age, or (ii) fat-free mass index (FFMI) <15 and 17 kg/m^2^ in women and men, respectively.

A few years later, in 2019, ESPEN, together with the Global Leadership Initiative on Malnutrition (GLIM), developed new diagnostic criteria for MN. They considered, in addition to phenotypic criteria similar to those previously published (low BMI, unintentional weight loss, and low muscle mass), etiological criteria, such as inflammation or disease burden, and reduced food intake or assimilation [11]. Different indicators, measurement techniques, and cut-off points to assess the loss of muscle mass were proposed: FFMI, appendicular skeletal muscle mass index (ASMMI), or calf circumference [11]. For etiological criteria, especially the one referring to inflammation or disease burden, both the clinical judgment of specialists and biochemical indicators (CRP, albumin, etc.) can be employed. Subsequently, other indicators of muscle mass loss were included, such as the skeletal muscle mass index (SMMI) [5].

Then, in 2022, in order to validate the usefulness of the GLIM criteria for predicting therapeutic response to nutritional intervention, Kaegi-Braun et al. performed a secondary analysis. They collected data from a multicenter randomized trial carried out between 2014 and 2018 in patients at risk of malnutrition who were prescribed two different nutritional therapies. Since this study was prior to the definition of the GLIM criteria, they did not have all the variables necessary for its application. As a result, they defined a modified GLIM criterion (mGLIM), where a reduction in handgrip strength (HS) was used as a phenotypic criterion since it is a proxy for reduced muscle mass [12]. This work demonstrated the strong prognostic value of mGLIM criteria regarding adverse clinical and other long-term outcomes.

Since the introduction of the GLIM criteria, numerous articles have been published assessing their validity or feasibility [13]. However, the number of studies conducted on institutionalized psychogeriatric patients remains limited. The prevalence of MN in the institutionalized geriatric population is notability high [14], particularly when these patients suffer from chronic pathologies, which are associated with inflammation and, in many cases, unfavorable body composition changes [15,16,17]. Furthermore, dementia itself is a documented risk factor for malnutrition. In this regard, a higher risk is observed in more advanced stages of the disease [8,18,19,20]. Schizophrenia is also a mental condition associated with an increased nutritional risk [21,22]. Early identification of malnutrition in these subjects is essential to initiate an appropriate and individual nutritional intervention to (1) reverse the situation, (2) improve functional capacity, and (3) reduce morbidity, mortality, and healthcare costs [3].

The bioimpedance analysis (BIA) is a technique with little technical difficulty; it is also harmless, portable, repeatable, precise, economical, and objective [23]. Hence, it is becoming increasingly popular in routine clinical practice due to its demonstrated validity in body composition analysis, and it is one of the methods endorsed by the GLIM Core Leadership Committee and GLIM Working Group for body composition analysis [11]. The phase angle (PhA) obtained from BIA reflects the relationship between resistance and reactance. It is an indicator of cell membrane integrity, thereby reflecting cellular mass [24], and it is regarded as a reliable indicator of cellular function, hydration status, and, consequently, nutritional status [25]. Its utility as a predictive factor for mortality and risk of complications in various pathologies has been demonstrated, as well as a prognostic factor in different types of cancer and an indicator of cellular survival [25,26]. Several studies have documented a relationship between PhA and muscle mass, strength, and function [27]. Since PhA is independent of predictive models, specific anthropometric measurements, or assumptions regarding morphology or hydration, it could be a highly valuable variable in clinical practice and in the nutritional assessment of older patients, where precise anthropometric measurements may not always be achievable [25].

The aim of this study was to analyze the diagnostic capacity of the GLIM criteria, using different indicators to assess the loss of muscle mass and the ESPEN criteria to diagnose malnutrition in a sample of institutionalized psychogeriatric patients.

## 2. Materials and Methods

A cross-sectional study was carried out in a psychogeriatric center in Palencia (Spain). The sample was formed by all the institutionalized subjects from the center who did not suffer from any acute intercurrent disease and who did not present any contraindication for bioimpedance analysis (water imbalances, amputations, metallic prostheses, or pacemakers). Approval was obtained from the Clinical Research Ethics Committee (CEIC) of Valladolid-East Health Area (protocol code: PI 22-2632 (10 March 2022)).

Clinical and demographic data were extracted from the medical records. The main pathology was diagnosed according to the *Diagnostic and Statistical Manual of Mental Disorders, Fifth Edition* (DSM-5) [28] and the *International Classification of Diseases, 11th Revision* (ICD-11) [29]. Comorbidity was assessed with the Charlson Comorbidity test [30]. Polypharmacy was defined as the concurrent use of five or more drugs per day [31]. Frailty was assessed using the FRAIL test [32], and dependence using the Barthel index [33]. Functional assessment was determined with the Time Up and Go test [34]. Handgrip strength was measured with a hydraulic hand dynamometer (Jamar, Nottinghamshire, UK) following the American Society of Hand Therapists (ASHT) protocol [35]. The diagnosis of sarcopenia was established according to the revised European Working Group on Sarcopenia in Older People (EWGSOP2) diagnostic criteria [36]. Malnutrition screening was performed by applying the full Mini-Nutritional Assessment (MNA) [37].

Anthropometric determinations (weight, height or knee-hell length, and body circumferences) were performed according to NHANES [38] and WHO [39] protocols and by using conventional methods (a scale and vertical stadiometer (SECA, Hamburg, Germany), and a non-stretchable tape measure (Cescorf, Porto Alegre, Brasil)). Nutritional assessment was carried out following the protocol of the Spanish Society of Parenteral and Enteral Nutrition (SENPE) and the Spanish Society of Geriatrics and Gerontology (SEGG) [40]. BMI was cataloged according to the WHO cut-off points [41] and the consensus document for the elderly population [39]. Waist circumference was also cataloged according to the WHO cut-off points [42].

Whole-body BIA was performed with a tetrapolar electrode configuration in single-frequency mode at 50 kHz with a BIA-101 analyzer (Akern, Srl, Florence, Italy), following the standard protocol [43]. Raw electrical variables [resistance (R), reactance (Xc), and phase angle (PhA)] were collected and the following body compartments were estimated: fat-free mass (FFM) using the model of Kyle et al. [28] and its reference values for the European Caucasian population [44]; and skeletal muscle mass (SMM), using the Janssen equation [45] and appendicular skeletal muscle mass (ASM), using the model of Sergi [46]. FFM, ASMM, and SMM index were calculated by dividing those compartments by squared height (in m) (FFMI, ASMMI, and SMMI). Muscle quality was assessed from the muscle quality index (MQI) (maximum handgrip strength/skeletal muscle mass) [47].

The diagnosis of malnutrition was established using the European Society for Clinical Nutrition and Metabolism (ESPEN) criteria [10], the GLIM criteria based on the cut-off points established for FFMI, ASMMI, and SMMI (GLIM 1, GLIM 2, and GLIM 3, respectively) [5,11,48], and the recently published modified GLIM criteria (mGLIM) [12]. The CRP value recommended by ESPEN [49] was used for all GLIM criteria as the cut-off point for inflammation.

Categorical variables are described as absolute (n) and relative frequency (%), and quantitative variables are described as mean (SD). The normality of the variables was determined using the Kolmogorov–Smirnov test. Differences between the different quantitative variables based on a dichotomous variable were analyzed with the Student’s t-test for independent measures or the Mann–Whitney U test (nonparametric measures). Differences between categorical variables were analyzed using the chi-square test. Agreement between the different methods was assessed using Cohen’s Kappa index. Statistical significance was reached with *p* < 0.05. Statistical analysis was performed with the SPSS 29.0 statistical package for Windows.

## 3. Results

The sample of the study was formed by 92 patients (53 men (57.6%) and 39 women (42.4%)), with a mean age of 79.4 years (SD: 10.2; range: 56.3 to 105.5). The average length of stay in the center was 15.9 years (SD: 20.2; range: 0.3 to 77.1 years).

A total of 52.2% of the sample presented dementia diagnosis (major cognitive disorder), 19.6% schizophrenia, 18.5% intellectual disability, and 9.8% other psychiatric pathologies. According to the Charlson index, the majority of subjects presented high comorbidity (62% high comorbidity, 35.9% low comorbidity, and 2.2% no comorbidity). Most subjects (97.8%) used three or more drugs per day (polypharmacy). The most frequent pathologies were chronic respiratory pathology (83.7%), cardiovascular pathology (37%), kidney pathology (26.1%), oncological pathology (17.4%), congestive heart failure (8.7%), and liver pathology (2.2%).

According to the Barthel classification, all the sample was dependent: 20.7% totally dependent, 46.7% severely dependent, 27.2% moderately dependent, and 5.4% mildly dependent. Regarding frailty, the Frail test only detected 6.5% of the patients as robust, 35.9% as pre-fragile, and 57.6% as fragile. The Time Up and Go test reflected reduced values in 89.1% of the patients.

Anthropometric and body composition variables are summarized in Table 1. According to BMI, 20.7% of the sample were undernourished, 25% were underweighted, 45.7% presented nutritional normality, 4.3% were overweight, and the remaining 4.3% were obese. A total of 39.1% of the subjects were at high risk of metabolic complications according to waist circumference. Body composition estimated by conventional BIA showed reduced FFM (<P5 in males and P5–P10 in females), with preserved fat mass (P25–P50 in both sexes). ASMMI reflected a reduced ASMM in both men and women. All body composition variables were statistically different according to sex (*p* < 0.001).

The mean maximum handgrip strength was 9.5 (10.0) kg in men and 5.7 (6.1) kg in women (*p* = 0.027). A total of 95.7% of patients had reduced handgrip strength, according to EWGSOP2 criteria. The mean muscle quality index was 0.56 (0.58) in men and 0.43 (0.48) in women, with no significant differences by sex. All women and 96.2% of men presented poor muscle quality. Just two male subjects presented normal (1.9%) or reduced (1.9%) muscle quality. Finally, the EWGSOP2 criteria were applied, and it was observed that 72.8% of the patients had sarcopenia.

The mean score of the MNA was 16.8 points (4.1). According to this screening scale, all patients, except one, were identified as malnourished (44.6%) or at risk of malnutrition (54.3%).

The prevalence of malnutrition in the sample was 25% applying the ESPEN criteria, 33.7% using the GLIM criteria with the FFM-I (GLIM 1), 38% using the GLIM criteria with the ASMM-I (GLIM 2), 41.3% when the GLIM criteria with the SMM-I (GLIM 3) were applied, and 40.2% using the modified GLIM criteria (mGLIM).

Table 2 reflects the characteristics of patients with malnutrition (MN) and with non-malnutrition (non-MN) according to the different diagnostic criteria used. The ESPEN criteria showed that malnourished subjects presented lower statistically significant values of anthropometric and body composition variables (except for SMMI), albumin, and MNA scores. GLIM 1 criteria discerned significant differences between malnourished and non-malnourished subjects in BMI, FFMI, FMI, and ASMMI index. These criteria also distinguished these differences in biochemical indicators (albumin and CRP), MNA score, and phase angle. However, the GLIM 2 criteria only differentiated subjects in a statistically significant way according to BMI, FMI, CRP, and MNA scores, but they failed to differentiate any muscle mass compartment. GLIM 3 criteria only found significant differences between MN and non-MN subjects in the mean CRP value, while mGLIM criteria only discriminated on muscle quality index, MNA score, and CRP.

No statistically significant differences were found in main pathology, dependence, frailty, and functional capacity between MN and non-MN subjects, regardless of the diagnostic criteria used. There were significantly more MN men when they were classified with GLIM2 (*p* = 0.036) and mGLIM (*p* = 0.044) criteria, but not with the rest of them. There was also a greater number of subjects that registered an unintentional weight loss of >10% among MN patients classified with the ESPEN criteria compared to the non-MN ones (this difference was not observed with the rest of the diagnostic criteria). All GLIM criteria showed that in the group of MN subjects, there was a greater number of patients with a clinical judgment of inflammation than in the non-MN group.

The agreement in the classification of subjects (MN and non-MN) using the different diagnostic criteria was evaluated (Table 3). The observed agreement between the ESPEN criteria and all GLIM criteria was poor (kappa < 0.2). However, the agreement between all GLIM criteria using different indicators to assess muscle mass loss was excellent (kappa > 0.8).

Finally, Table 4 shows the prevalence of etiological and phenotypic criteria considered to identify malnutrition according to the GLIM and ESPEN initiatives. Phenotypic criteria carried more weight in the diagnosis of MN than etiological ones. On the one hand, according to the variables of the etiological criteria, in our sample, the objective criterion of inflammation (CRP) identified a greater number of patients at risk of MN than the clinical judgment of the specialist. On the other hand, regarding the variables of the phenotypic criteria, important differences were shown in the prevalence of reduced muscle mass depending on the parameter used for its detection: handgrip strength, ASMMI, and, above all, SMMI identified a greater MN prevalence than FFMI and BMI.

## 4. Discussion

The definition of malnutrition has evolved in recent years. Currently, it is defined as “a subacute or chronic state of nutrition, in which a combination of varying degrees of under- or overnutrition and inflammatory activity has led to changes in body composition and diminished function” [50], which reflects the importance of inflammation and changes in body composition. As a consequence, objective, simple, and easy-to-apply diagnostic criteria have been proposed considering not only the phenotypic characteristics of malnutrition (weight, BMI, and muscle mass), as the ESPEN criteria do [10], but also the etiological component. Thus, a consensus was published for the diagnosis of malnutrition based on the application of a set of operational criteria (the GLIM criteria) that subsequently needed to be validated [11]. The cut-off points for the phenotypic criteria of these initial GLIM initiatives were established based on the available scientific evidence [50]. But, since they were conceived as a tool for global use, the same criterion could be assessed with different indicators or variables and measurement methods which depend on the following: (1) available resources and (2) threshold values (being different according to ethnicity or sex) [51]. Therefore, it could be expected that the validity of the GLIM criteria for predicting malnutrition and its related complications would be affected by the use of different indicators and methods [51]. In this sense, it has also been documented that the inclusion of different indicators (determined by different methods) provided very different estimations of the prevalence of reduced muscle mass [52]. Moreover, several studies, regardless of indicating that they followed the GLIM initiative, did not apply all phenotypic criteria (generally excluding muscle mass loss), and so, a much lower prevalence of MN than expected was provided [53].

Related to the etiological variables, clear objective criteria were also not defined because of a lack of consensus on precise markers and cut-off points [50]. In the case of the “presence of inflammation or disease burden” criterion, it is considered that burns and trauma will cause states of acute inflammation, whereas most chronic diseases such as congestive heart failure, chronic obstructive pulmonary disease, rheumatoid arthritis, chronic kidney or liver disease, and cancer, are associated with chronic inflammation. The GLIM criteria also identify a positive inflammation in cases of fever, negative nitrogen balance, and high resting energy expenditure [51]. Therefore, although the identification of the presence of inflammation or disease burden can be a rather subjective criterion in many cases, the underlying medical diagnosis has been considered valid for assessment in clinical settings [50]. The presence of inflammation can also be established from the determination of some laboratory indicators, such as serum C-reactive protein, serum albumin, or prealbumin [51]. In fact, it is recommended to use these biochemical indicators of inflammation when aiming to validate the GLIM criteria [50].

In a very aged population, like our sample, where comorbidity is present in 97.9% of the patients (basically from chronic pathologies), the clinical judgment of inflammation, a priori, is always going to be positive. Thus, it was decided to use an objective biochemical indicator commonly used as a parameter of inflammation, such as CRP. Given the characteristics of our study population, although the threshold value for this marker has not been clearly defined [12,54], it was decided to follow the ESPEN recommendations [49] and use a value of ≥5 mg/L to identify the presence of inflammation.

The aim of this study was not to validate the GLIM criteria but to determine which criteria (or which variables) could help to better identify malnutrition in a sample of psychogeriatric patients with high cognitive impairment, frailty, and dependence. Some authors have questioned whether screening is mandatory before employing GLIM criteria [55]. In our case, since all except one of the subjects included in the study were categorized as malnourished or at risk of malnutrition according to the MNA, it was decided to apply the GLIM criteria to the entire group.

Depending on the diagnostic criteria used, in the present work, the prevalence of MN observed varied between 25% (ESPEN criteria) and 41.3% (GLIM3 criteria, with the SMMI). A recent review of 40 studies conducted between 2021 and 2022 [13] found prevalence values of malnutrition (identified with the GLIM initiative) from 11% in community-dwelling elderly subjects to 74% in patients with hip fractures or even 95% in geriatric patients in rehabilitation. Similarly, in a meta-analysis in which 20 studies were evaluated, with a total population of 10,781 patients, where the aim was to determine the accuracy of the GLIM criteria for the diagnosis of malnutrition [56], a mean prevalence of MN of 44.2% was reported (ranging from 11%—in a large sample of Japanese subjects with cardiovascular disease—to 90.1%—in a group of hospitalized elderly Italians). It should be highlighted that despite all the studies included in both publications being performed with very different populations (community-dwelling elderly subjects, hospitalized patients, patients with cancer and other pathologies, geriatric rehabilitation patients, ICU patients, etc.), all of them registered a higher prevalence of MN in institutionalized and hospitalized patients than in people living in the community. There are also differences in the prevalence of MN according to functional status, cognitive impairment, mobility limitations, and dependence [4]. Furthermore, there is huge variability in the criteria used for diagnosis, both phenotypic and etiological. This makes it extremely difficult to compare results.

Prevalence values of malnutrition identified with GLIM criteria documented in Caucasian geriatric populations range from 46.6% in a group of 60 Swiss geriatric rehabilitation patients with GLIM1 and CRP > 10 mg/L [57] to 64% in Swedish geriatric patients with GLIM1 criteria [58]. Our results are comparable to those reported in the work of Ohta et al., where similar criteria to ours were used. A prevalence of 11.2% has been documented in a sample of 464 Spanish subjects living in three nursing homes [59] with GLIM1 criteria. This value is much lower than the one from our study and closer to those reported in community-dwelling older adults (from 11%—Asian populations—to 32%—European populations) [13]. It is noteworthy that the majority (97.6%) of the Spanish subjects in the study by González-Fernández et al. presented some degree of dependence, but they were not psychogeriatric patients nor with dementia. In this regard, a study that evaluated 65 Norwegian patients with mild dementia [60] found that 23.7% were malnourished, but for diagnosis, they only used BMI as a phenotypic criterion, and dementia was considered a positive etiological criterion in all patients.

Agreement in the diagnosis of MN established with the five criteria used in the study was poor between the ESPEN criteria and any of the GLIM criteria. Several studies of comparison and agreement of the GLIM criteria with other diagnostic methods have been performed [1,5,13], including the ESPEN criteria. In all of them, agreement between GLIM and ESPEN criteria was poor, and the prevalence of malnutrition using ESPEN criteria was much lower than with other criteria (such as GLIM), as observed in our study.

These differences are due to the inclusion of etiologic criteria to identify MN with the GLIM criteria (but not with the ESPEN criteria), especially inflammation. MN subjects diagnosed with any of the GLIM criteria used in the study have a significantly higher CRP value than non-malnourished subjects (Table 2). These differences were also observed when a less objective criterion was used in the consideration of inflammation, such as the physician’s clinical judgment. However, no differences in inflammation (determined by CRP or clinical judgment) were found between MN and non-MN subjects diagnosed with ESPEN criteria.

Nutritional status classification of subjects with the ESPEN criteria divides the sample into two groups with statistically significant differences in BMI, waist circumference, and all body composition variables (except SMMI). These differences are remarkable for anthropometric variables and FM indicators. Moreover, these are the only criteria by which the two groups differ in unintentional weight loss. Therefore, these criteria could be the best to reflect the phenotypic characteristics of malnutrition in these patients.

Similarly, the GLIM1 criteria (which include FFMI) also discern between malnourished subjects according to BMI, FFMI, FMI, and ASMMI (not to SMMI and waist circumference). Also, these criteria were the only ones that discriminated between malnourished and non-malnourished subjects according to phase angle. Several studies have documented a lower PhA in malnourished subjects [61]; in our work, it was only observed in patients classified as malnourished with the GLIM1 criteria. This could be due to the fact that all the patients in our study presented quite reduced PhA values (4.26° (0.72) in men and 3.82° (0.94) in women). A recent systematic review of 46 studies that included a population of 249,844 subjects established the following PhA reference values: (1) 70–80 years: 5.6 (95% CI: 4.8–6.4) in men, and 5.1 (95% CI: 4.7–5.5) in women; and (2) over 80 years: 5.3 (95% CI: 4.5–6.0) in men, and 5.4 (95% CI: 5.3–5.6) in women [62]. So, the mean PhA value of our sample was much lower than the inferior limit of 95% CI from the reference values. Despite this, this criterion identified significant differences in phase angle (PhA) value between MN and non-MN subjects. This lower PhA observed in MN subjects indicates that they presented lower FFM and SMM [25], representing a lower body cell mass, which is recognized as the most reliable predictor of nutritional status [26].

The GLIM2 criteria (employing the ASMMI) regarding body composition only differentiated MN from non-MN subjects according to BMI and FFM. However, the cut-off points recommended in the GLIM initiative consensus [11,36] that were used were established from a young reference population (20–39 years) [63]. So, it is possible that these are very strict cut-off points for our study population. It could be necessary to identify specific cut-off points for the older population that truly reflect a reduction in muscle mass.

The GLIM3 criteria (which employ the SMMI) only discern between MN and non-MN subjects according to the CRP value, but not for any of the phenotypic variables. This indicator was part of the diagnostic criteria of the first sarcopenia consensus [64], but it was replaced by ASMMI in the second consensus [36]. The SMMI cut-off points were obtained from a population over 60 years [48]; since 85.9% of our participants had an SMMI below the cut-off, they also seem quite strict for an aged population like ours. This indicator does not discriminate between groups (MN vs. non-MN) by body composition.

Finally, the mGLIM criteria, which use handgrip strength as an indicator of muscle mass loss, only show statistically significant differentiation between the two groups of subjects (MN vs. non-MN) in the muscle quality index (in addition to CRP, as mentioned above) and the MNA score. Since the muscle quality index is calculated as maximum handgrip strength/skeletal muscle mass, this result is expected [47]. Our data seem to indicate that, in a psychogeriatric population with similar characteristics to ours (high cognitive impairment, dependence, frailty, and reduced functional capacity), handgrip strength does not reflect the loss of muscle mass. The great majority of our participants (95.7%) presented reduced handgrip strength, according to the EWGSOP2 criteria. In fact, in many cases (33.7%), grip strength was considered zero when patients were unable to hold the dynamometer or, more frequently, to understand the instructions for taking the measurement. Thus, handgrip strength may not be useful as an indicator of muscle strength and/or function in subjects with significant cognitive impairment. In that case, the mGLIM criteria would not be suitable as diagnostic criteria for malnutrition.

In our study, statistically significant lower plasma albumin values were observed in MN patients diagnosed with the ESPEN and GLIM 1 criteria. So, those criteria could differentiate MN from non-malnourished patients according to muscle mass (independently from CRP). Several studies have documented that reduced albumin levels in older people are associated with loss of muscle mass [65,66], poor physical performance [67], increased disability [66], and decreased muscle strength [66,67]. Subjects with sarcopenia also have lower serum albumin values than non-sarcopenic subjects [65].

Regardless of the criteria used, mean albumin values in our study are very low in both MN subjects (<3.0 g/dL) and non-MN (<3.5 g/dL). Although some authors have also reported values similar to ours [68,69], our results are lower than the majority of the values published in previous articles [65,66,68]. Serum albumin concentration has been widely used as an indicator of nutritional status (cut-off point for MN: <3.5 g/dL serum albumin) [66]. However, several authors have questioned both (1) the usefulness of albumin as an indicator of malnutrition in the elderly population since this protein is also affected by inflammatory and infectious processes and various diseases and alterations in hydration status [65,66,69,70] and (2) the value of 3.5 g/dl as a cut-off point for MN [69]. Some authors conclude that albumin is only a useful indicator of MN in cases of clinical stability [66]. Our sample presents high comorbidity, dependence, and frailty, which could explain the reduced albumin levels observed. In addition, more than half of the subjects have dementia with great cognitive impairment. Some studies have reported reduced serum albumin levels in patients with different types of dementia [71].

The MNA score was significantly lower in MN patients compared to the non-MN ones identified using all the criteria studied, except for GLIM3. All subjects, except one, were classified as MN or at risk of MN, according to MNA. Since in all MN subjects (identified using any of the evaluated criteria) the average MNA score was below 17 points, our results reflect the strength of this questionnaire as a MN screening tool.

The different criteria for the diagnosis of MN analyzed (Table 4) showed that inflammation is the most prevalent etiological criterion in our sample. Considering the type of population being evaluated, this result is an expected finding. The aging process involves chronic low-grade inflammation, and additionally, the capacity for liver protein synthesis is decreased. It should be noted that frailty implies a difficulty in adaptation, with a reduced capacity for physiological response to any stimulus. Therefore, in older patients with multiple comorbidities, does it make sense to use disease burden and/or the presence of inflammation as etiological criteria?

Regarding phenotypic criteria, a low BMI is one of the most frequent in the sample studied. A review of 11 studies evaluating nearly 6000 older adults in different settings across 10 European countries and New Zealand revealed a high prevalence of low BMI in the elderly population with dependence and frailty (especially in nursing home residents) and a reduced frequency of unintentional weight loss [4], as shown in our study. Given that significant weight loss was less prevalent in the older age group (the group with the highest prevalence of low BMI), evidence suggests that the application of the ESPEN criteria to certain populations could underestimate the presence of MN [4]. It seems that the rate of weight loss slows with age and that, in subjects who have been institutionalized for a long time, weight loss occurs at earlier ages, reflecting a lower BMI in older age [4]. Loss of strength and muscle mass are also very prevalent, especially when it is assessed according to SMMI or ASMMI, which coincides with the diagnosis of sarcopenia observed (72.8%—EWGSOP2 criteria). In these subjects, where, in addition to what has already been mentioned, physical activity is very reduced, it is possible that FFM reflects the nutritional status better than SMM or ASMM

Therefore, based on the results obtained, we consider that for diagnosing MN in institutionalized psychogeriatric patients with high comorbidity, disability, and frailty, the GLIM criteria, which include the FFMI indicator to assess the loss of muscle mass (GLIM1), are the most appropriate. These criteria significantly discern between MN and non-MN subjects according to both BMI and body composition, as well as in the presence of inflammation (CRP) and albumin levels. Moreover, they are the only criteria that distinguish between the two groups (MN and non-MN) according to the phase angle value. Applying a quick, simple, and direct indicator unaffected by predictive models, such as the PhA, could be very useful in monitoring the nutritional status of this population.

This study presents some limitations, including the small sample size and, especially, those derived from the characteristics of the sample studied, which primarily consists of long-term institutionalized subjects who are highly deteriorated, with high cognitive impairment, comorbidity, dependence, and frailty. Consequently, it could be difficult to generalize the results to other elderly populations in different care settings. Additionally, muscle mass was estimated using bioelectric impedance, a technique that may not be accurate if the assumptions of water–electrolyte balance are not met [72]. In this sense, subjects who might present problems of edema or dehydration were excluded. We propose the utility of a simple indicator unaffected by the aforementioned assumptions, such as the phase angle. Some strengths of this study include the application of diagnostic criteria that are easily evaluable in routine clinical practice without requiring expensive and difficult-to-use equipment. Furthermore, since the studied population is institutionalized, the variables were prospectively collected by specialized personnel, ensuring the quality of the data.

Several longitudinal studies have shown an elevated risk of mortality in patients identified as MN using the GLIM criteria [13,73]. The follow-up of our sample over time will enable us to determine the predictive ability for mortality of the different diagnostic criteria evaluated in this study, both individually and as components of different algorithms, in the psychogeriatric population. It will also enable us to evaluate their utility and that of the phase angle as predictors of response to nutritional interventions.

## 5. Conclusions

GLIM1 criteria, which include the FFMI indicator to assess the loss of muscle mass, show differences in BMI, body composition, inflammation, and albumin between MN and non-MN subjects. Additionally, this criterion is the only one that identified differences between these two groups according to phase angle value, which can be particularly useful in monitoring the nutritional status of this population.

## Figures and Tables

**Table 1:** Anthropometric and body composition variables of the sample (mean (SD)).

Variables	Total Sample	Men	Women
BMI (kg/m^2^)	22.5 (4.7)	22.5 (3.9)	22.5 (5.6)
WC (cm)	93.3 (10.4)	95.0 (9.1)	91.0 (11.6)
FFM (kg)	39.8 (8.0)	44.4 (5.9)	34.0 (6.6) *
FM (kg)	16.7 (8.4)	14.7 (7.8)	19.7 (8.4) *
FFMI (kg/m^2^)	15.7 (2.4)	16.8 (1.7)	14.3 (2.4) *
FMI (kg/m^2^)	6.8 (3.5)	5.6 (2.9)	8.3 (3.6) *
ASMM (kg)	15.0 (3.3)	16.7 (2.5)	12.6 (2.8) *
ASMMI (kg/m^2^)	5.9 (1.0)	6.3 (0.7)	5.3 (1.0) *
SMM (kg)	19.5 (5.6)	23.0 (3.8)	14.7 (3.9) *
SMMI (kg/m^2^)	7.6 (1.7)	8.7 (1.1)	6.2 (1.4) *
BMI (kg/m^2^)	22.5 (4.7)	22.5 (3.9)	22.5 (5.6)
WC (cm)	93.3 (10.4)	95.0 (9.1)	91.0 (11.6)
FFM (kg)	39.8 (8.0)	44.4 (5.9)	34.0 (6.6) *
FM (kg)	16.7 (8.4)	14.7 (7.8)	19.7 (8.4) *
FFMI (kg/m^2^)	15.7 (2.4)	16.8 (1.7)	14.3 (2.4) *
FMI (kg/m^2^)	6.8 (3.5)	5.6 (2.9)	8.3 (3.6) *
ASMM (kg)	15.0 (3.3)	16.7 (2.5)	12.6 (2.8) *
SMM (kg)	19.5 (5.6)	23.0 (3.8)	14.7 (3.9) *
SMMI (kg/m^2^)	7.6 (1.7)	8.7 (1.1)	6.2 (1.4) *

* *p* < 0.05. BMI—body mass index; WC—waist circumference; FFM—fat-free mass; FM—fat mass; FFMI—fat-free mass index; FMI—fat mass index; ASMM—appendicular skeletal muscle mass; ASMMI—appendicular skeletal muscle mass index; SMM—skeletal muscle mass; SMMI—skeletal muscle mass index.

**Table 2:** Characteristics of malnourished and non-malnourished patients classified according to diagnostic criteria (mean (SD)).

Variables	ESPEN		GLIM 1 (FFMI)		GLIM 2 (ASMMI)		GLIM 3 (SMMI)		mGLIM	
	MN (*n* = 23)	Non-MN (*n* = 71)	MN (*n* = 31)	Non-MN (*n* = 61)	MN (*n* = 35)	Non-MN (*n* = 57)	MN (*n* = 38)	Non-MN (*n* = 54)	MN (*n* = 37)	Non-MN (*n* = 55)
Age (years.)	78.4 (10.5)	79.7 (9.9)	78.6 (10.8)	79.0 (10.5)	80.5 (9.1)	79.6 (10.1)	79.0 (10.2)	79.6 (10.2)	78.7 (10.2)	79.8 (10.2)
LOS (years)	10.8 (14.5)	17.8 (21.6)	10.9 (15.7)	18.5 (21.8) *	12.4 (17.4)	18.1 (21.6)	12.5 (16.8)	18.3 (22.0)	12.7 (16.9)	18.1 (21.9)
BMI (kg/m^2^)	18.1 (2.2)	24.0 (4.3) *	20.8 (3.5)	23.4 (4.9) *	21.2 (3.7)	23.3 (5.0) *	21.7 (3.9)	23.1 (5.1)	21.7 (4.0)	23.1 (5.0)
WC (cm)	86.3 (9.5)	95.7 (9.6) *	91.6 (9.0)	94.2 (11.0)	92.3 (10.0)	93.9 (10.7)	92.9 (9.9)	93.6 (10.8)	92.9 (10.1)	93.6 (10.7)
FFM (kg)	36.0 (6.4)	41.3 (8.2) *	40.0 (7.4)	39.7 (8.4)	40.9 (7.4)	39.2 (8.4)	40.5 (7.5)	39.4 (8.4)	40.7 (7.5)	39.2 (8.4)
FFMI (kg/m^2^)	14.2 (1.9)	16.3 (2.3) *	15.0 (2.1)	16.1 (2.4) *	15.4 (2.2)	15.9 (2.4)	15.5 (2.2)	15.9 (2.5)	15.6 (2.3)	15.9 (2.5)
FM (kg)	9.7 (5,7)	19.1 (7.8) *	15,1 (7.5)	17.5 (8.5)	15.3 (7.9)	17.6 (8.6)	16.0 (8.0)	17.3 (8.7)	15.8 (8.0)	17.4 (8.6)
FMI (kg/m^2^)	3.9 (2.5)	7.7 (3.3) *	5.8 (2.8)	7.3 (3.7) *	5.8 (2.9)	7.4 (3.8) *	6.2 (3.1)	7.2 (3.8)	6.1 (3.1)	7.2 (3.7)
ASMM (kg)	13.4 (2.6)	15.5 (3.6) *	15.0 (3.0)	14.9 (3.4)	15.3 (3.0)	14.7 (3.5)	15.2 (3.0)	14.8 (3.5)	15.3 (3.0)	14.7 (3.5)
ASMMI (kg/m^2^)	5.3 (0.8)	6.1 (0.9) *	5.6 (0.9)	6.0 (1.0) *	5.8 (0.9)	6.0 (1.0)	5.8 (0.9)	6.0 (1.0)	5.8 (0.9)	6.0 (1.0)
SMM (kg)	18.3 (5.3)	20.0 (5.7) *	20.0 (5.6)	19.2 (5.6)	20.6 (5.3)	18.8 (5.7)	20.2 (5.5)	19.0 (5.7)	20.4 (5.4)	18.9 (5.7)
SMMI (kg/m^2^)	7.1 (1.8)	7.8 (1.7)	7.5 (1.8)	7.7 (1.7)	7.8 (1.7)	7.6 (1.7)	7.7 (1.8)	7.6 (1.7)	7.8 (1.7)	7.6 (1.7)
MQI	0.31 (0.49)	0.43 (0.39)	0.30 (0.38)	0.45 (0.43)	0.30 (0.37)	0.46 (0.44)	0.30 (0.36)	0.46 (0.45)	0.29 (0.35)	0.47 (0.45) *
HS (kg)	5.7 (9.1)	8.7 (8.6)	6.6 (9.1)	8.5 (8.6)	6.7 (8.7)	8.6 (8.8)	6.6 (8.4)	8.8 (8.9)	6.5 (8.5)	8.8 (8.8)
Albumin (g/dL)	2.73 (0.36)	3.16 (0.36) *	2.87 (0.40)	3.13 (0.38) *	2.96 (0.45)	3.10 (0.37)	2.96 (0.44)	3.11 (0.37)	2.96 (0.44)	3.11 (0.37)
PCR (mg/L)	5.84 (4.66)	7.43 (12.9)	15.56 (16.2)	2.95 (3.9) *	14.6 (15.3)	2.37 (3.2) *	14.2 (14.8)	2.02 (2.9) *	13.4 (14.2)	2.74 (6.1) *
MNA (points)	13.3 (4.0)	17.9 (3.4) *	15.4 (4.3)	17.5 (3.8) *	15.7 (4.3)	17.5 (3.8) *	16.0 (4.3)	17.4 (3.8)	16.0 (4.4)	17.4 (3.8) *
PhA (º)	4.06 (1.08)	4.06 (0.76)	3.86 (0.63)	4.19 (0.93) *	3.87 (0.63)	4.20 (0.94)	3.91 (0.63)	4.19 (0.96)	3.91 (0.63)	4.18 (0.96)

* *p* < 0.05. MN—malnourished; non-MN—non-malnourished; LOS—length of stay; BMI—body mass index; WC—waist circumference; FFM—fat-free mass; FM—fat mass; FFMI—fat-free mass index; FMI—fat mass index; ASMM—appendicular skeletal muscle mass; ASMMI—appendicular skeletal muscle mass index; SMM—skeletal muscle mass; SMMI—skeletal muscle mass index; MQI—muscle quality index; HS—hand grip strength; PhA—phase angle.

**Table 3:** Agreement in the classification of subjects (malnourished vs. non-malnourished) with the different diagnostic criteria evaluated.

Cohen’s Kappa Coefficient	ESPEN	GLIM 1 (FFMI)	GLIM 2 (ASMMI)	GLIM 3 (SMMI)	mGLIM
ESPEN	1.000	0.169	0.111	0.071	0.084
GLIM 1 (FFMI)	0.169	1.000	**0.859**	**0.839**	**0.814**
GLIM 2 (ASMMI)	0.111	**0.859**	1.000	**0.932**	**0.909**
GLIM 3 (SMMI)	0.071	**0.839**	**0.932**	1.000	**0.977**
mGLIM	0.084	**0.814**	**0.909**	**0.977**	1.000

In bold the statistically significant kappa coefficients (*p* < 0.05).

**Table 4:** Prevalence of the different etiological and phenotypic criteria considered by the GLIM and ESPEN criteria for the identification of malnutrition.

Criteria	Variables		Prevalence [*n* (%)]
Etiological	Reduced food intake (>50%)		9 (9.8)
	Inflammation	Clinical judgment of specialists	23 (25.0)
		CRP > 5 mg/L	37 (40.2)
Phenotypic	UWL > 10% over the last 6 months		10 (10.9)
	BMI	Very low (<18.5 kg/m^2^)	19 (20.7)
		Low (<20 kg/m^2^ if <70 years, or <22 kg/m^2^ if ≥70 years)	40 (43.5)
	Low MM	FFMI	56 (60.9)
		ASMMI	69 (75.0)
		SMMI	79 (85.9)
	Low HS		66 (71.7)

UWL—unintentional weight loss; BMI—body mass index; MM—muscle mass; FFMI—fat-free mass index; FMI—fat mass index; ASMMI—appendicular skeletal muscle mass index; SMMI—skeletal muscle mass index; HS—hand grip strength.

## Data Availability

Data are contained within the article.
